# An Essay on the Unity of Disease

**Published:** 1853-09

**Authors:** 


*An Essay on the Unity of Disease.* Read before the Alabama State Medical Association. By H. Backus, M. D.

This is the most intelligible development of the "Williardian" theory of the circulation, we have yet seen; albeit Dr. Backus does not mention, and probably had not heard of this *femme sage.* We shall attempt to induct our readers into the ideas, so ingeniously evolved by Dr. Backus. Starting with the proposition that molecular motion is necessary to molecular aggregation, or disintegration, he makes the pressure upon each other of molecules of different densities, the sourse of motion. He then quotes from the "Chemistry of Plants," by Dr. Draper: "Whenever the admission of oxygen into the air cells of the lungs is prevented, the circulation through them simultaneously stops. * * * What now, is the cause of that asphyxiated condition? Why does the blood cease to flow? * * * the *oxydation* of that blood, is the very cause of its movement. It is the *pressure* of the *deoxydized*, upon the *oxydized* blood, that drives the latter along the pulmonary veins to the heart. But should anything intervene to prevent that oxydation taking place, *no pressure*, and therefore, *no movement*, can ensue.

"It is the *pressure* of these different media of different densities upon each othes then, that that is *the* cause of the movement, or circulation of blood."

The reader will perceive in these extracts, that an action similar to that known as *displacement* is regarded by the author, as the *primum mobile* of the circulation. That the heart becomes a secondary organ, or rather one of a succession of hearts. Giving to the word heart the meaning of a circulatory propulsive force, we have many hearts. The lung is (in the order of time,) the first of these; the heart itself the second; the arterial contractibility the third; the draught of the capillary vessels the fourth; the vacuum occasioned by molecular disintegration, and removal, the fifth; and the external pressure of the atmosphere the sixth. All these, however, are only aids to that molecular motion, which in and of itself, is in the lower animals, and vermin, sufficient to maintain circulation. A reference to Mattevcci's experiments on endosmose and exosmose, would have aided the author in proving that atomic circulation, depends only on the form of its tubes, and not on a *vis a tergo.*

From this the author derives his theory of the unity of disease—an unity not of name, or symptom, or cause, but of phenomenon. "We affirm that wherever there is *disease*, there is a *common state*, or *condition*, differing only in degree." * * * "Disease is obstructed circulation, a disturbance of the fundamental condition laid down in our first proposition; a loss of equilibrium in the action, resistance, pressure of the different parts, solids, and fluids upon each other and the surrounding media." Before stating that "reaction implies a state of previous prostration, to react from," he attempts to sustain these three propositions:

1st. That prostration is the antecedent, the fulcrum of reaction.

2d. That this state of prostration is the only essential condition in disease; and

3d. That whatever reaction contributes to disease, is the same in kind with its antecedent.

The following quotation embodies the essential idea of Dr. B's theory:

"We have said that the conditions of motion, whether of molecules or masses, were media of different densities acting upon each other; that molecular motion was the type, the antecedent, the base of the motion of the mass; that these conditions were *common*, not only wherever there was motion, but form, or phenomenon, as heat; that sensation, common or special, was elicited solely by pressure, by molecular touch; that disease or death was obstructed circulation."

The essential and *common* feature of all disease—its unit—is *prostration;* which being interpreted, means *congestion.* By a different rationale, we had ourselves arrived at a similar conclusion. In an article on Spinal Irritation, published in December, 1851, in this Journal, we considered congestion as the pathology of that, and other neuralgic diseases; and again in the Journal for November, 1852, may be found an article on a "case of eccentric convulsions," which expresses more definitely the same idea, viz: that congestion was unvaryingly present, wherever disordered sensation, pain, chill, *or its pronounced form convulsions*, were manifested. We did not, however, allude to the molecular theory, for the reason, (a very good one,) that we were then ignorant of it, and we are not as yet prepared to adopt it.

Dr. Backus writes in a very condensed and lucid style, and whatever conclusions we may adopt, the careful perusal of this little essay, (of only a dozen pages,) will well repay itself, in its suggestion, if not in its direct influence. It is possible that in a future number we may speak of this matter in connection with the phenomena of nervous disease, and its relations to zymosis.

